# Transannular patch repair of tetralogy of Fallot with or without monocusp valve reconstruction: a meta-analysis

**DOI:** 10.1186/s12893-022-01474-6

**Published:** 2022-01-16

**Authors:** Xiaodong Wei, Tiange Li, Yunfei Ling, Zheng Chai, Zhongze Cao, Kerun Chen, Yongjun Qian

**Affiliations:** 1 Department of Cardiovascular Surgery, Hubei Minda Hospital of Hubei, Minzu University, Enshi, Hubei province China; 2 Department of Cardiovascular Surgery, West China Hospital, Sichuan University, No. 37 GuoXue Xiang, Chengdu, Sichuan 610041 People’s Republic of China

**Keywords:** Tetralogy of Fallot, Transannular patch, Monocusp valve

## Abstract

**Background:**

Tetralogy of Fallot (TOF) is one of the most common cyanotic congenital heart diseases. Pulmonary regurgitation is the most common and severe comorbidity after transannular patch (TAP) repair of TOF patients. It has not been confirmed whether a TAP repair with monocusp valve reconstruction would benefit TOF patients in perioperative period compared to those without monocusp valve reconstruction. The purpose of the study is to review and analyze all clinical studies that have compared perioperative outcomes of TOF patients undergoing TAP repair with or without monocusp valve reconstruction and conduct a preferable surgery.

**Methods:**

Eligible studies were identified by searching the electronic databases. The year of publication of studies was restricted from 2000 till present. The primary outcome was perioperative mortality, and secondary outcomes included cardiopulmonary bypass time, aortic cross-clamp time, ventilation duration, ICU length of stay, hospital length of stay, perioperative right ventricular outflow tract (RVOT) pressure gradient, and moderate or severe pulmonary regurgitation (PR). The meta-analysis and forest plots were drawn using Review Manager 5.3. Statistically significant was considered when *p-*value ≤ 0.05.

**Results:**

Eight studies were included which consisted of 8 retrospective cohort study and 2 randomized controlled trial. The 10 studies formed a pool of 526 TOF patients in total, in which are 300 undergoing TAP repair with monocusp valve reconstruction (monocusp group) compared to 226 undergoing TAP repair without monocusp valve reconstruction (non-monocusp group). It demonstrated no significant differences between two groups in perioperative mortality (OR = 0.69, 95% CI 0.20–2.41, *p* = 0.58). It demonstrated significant differences in perioperative cardiopulmonary bypass time (minute, 95% CI 17.93–28.42, *p* < 0.00001), mean length of ICU stay (day, 95% CI − 2.11–0.76, *p* < 0.0001), and the degree of perioperative PR (OR = 0.03, 95% CI 0.010.12, *p* < 0.00001). Significant differences were not found in other secondary outcomes.

**Conclusion:**

Transannular patch repair with monocusp valve reconstruction have significant advantages on decreasing length of ICU stay and reducing degree of PR for TOF patients. Large, multicenter, randomized, prospective studies which focuse on perioperative outcomes and postoperative differences based on long-term follow-up between TAP repair with and without monocusp valve reconstruction are needed.

**Supplementary Information:**

The online version contains supplementary material available at 10.1186/s12893-022-01474-6.

## Background

Tetralogy of Fallot (TOF) represents the most common form of a cyanotic congenital heart defect. It is associated with the morbidity of approximately 1/3500 in the newborns and accounts for 7% to 10% of all congenital cardiac malformations [1]. In TOF, there are four types of defects, including the overriding of the aorta, pulmonary stenosis or right ventricular outflow tract (RVOT) obstruction, ventricular septal defect (VSD), and right ventricular hypertrophy (RVH). A complete surgical repair to all these defects are commonly required, which consists of two main operations, namely enlarging the narrowed RVOT and closing the VSD [2].

Transannular patch (TAP) repair is one of the most effective approaches to enlarge the ROVT when the narrowed pulmonary annulus is insufficient to warrant a total correction [3]. This procedure includes an incision to the annulus of a malformed valve, and enlargement of the infundibulum and main pulmonary artery with a TAP [4], which is commonly performed when the RVOT is severely narrowed. The most obvious advantage of a TAP is that it can resolve right ventricular hypertension immediately [4]. However, it may also cause significant pulmonary regurgitation (PR) [5], leading to marked pulmonary incompetence and chronic delayed right ventricular dysfunction. Although a reoperation for ROVT obstruction is uncommon, late pulmonary valve insertion is rather frequent [6]. To prevent postoperative PR, a monocusp valve has been used in some practices [4, 7–9]. However, to date, monocusp valve reconstruction is not routinely practiced in TOF patients undergoing TAP repair for severe ROVT stenosis, given that the benefits of this approach comparing with non-monocusp valve reconstruction remains unclear. Our study aimed to systematically review and analyze all clinical studies that have compared perioperative outcomes of TOF patients undergoing TAP repair with or without monocusp valve reconstruction. The findings of this study would inform the best evidence-based practice in the field.

## Methods

### Perioperative outcomes

This meta-analysis was conducted to identify clinical studies that compared differences in the perioperative outcomes in TOF patients undergoing TAP repair with or without monocusp valve reconstruction. In our searching process, few relevant review articles or meta-analysis protocols were found. In our study, perioperative mortality was determined as the primary outcome. For secondary outcomes, continuous variables including cardiopulmonary bypass time, aortic cross-clamp time, ventilation duration, ICU length of stay, hospital length of stay, and perioperative RVOT pressure gradient were assessed. Dichotomous variables included moderate or severe pulmonary regurgitation in the perioperative period, besides perioperative mortality.

### Search strategy

Eligible studies were identified by searching the electronic databases including PubMed, Science Direct, Web of Science, Embase in Ovid, Cochrane Library and Scopus database. Detailed search strategies of PubMed: (1) ("Tetralogy of Fallot"[Mesh]) AND ((cusp) OR (monocusp) OR (monocuspid)), (2) (“Tetralogy of Fallot”[Mesh]) AND (transannular), (3) (pulmonary) AND ((cusp) OR (monocusp) OR (monocuspid)). Search strategies of Science Direct: “Tetralogy” AND “transannular” AND “monocusp”. Search strategies of Web of Science: “Tetralogy” AND “transannular” AND “monocusp”, searches were confirmed in “Advanced Search” and “Topic”. Search strategies of Embase through Ovid: “Tetralogy” AND “transannular” AND “monocusp” in “Advanced Search”. Search strategies of Cochrane Library: “Tetralogy” AND “transannular”, while searches were confirmed in “Advanced Search” and “Title Abstract Keyword”. Search strategies of Scopus: “Tetralogy” AND “transannular” AND “monocusp”, while searches were confirmed in “Documents” and “Article title, Abstract, Keywords” Literature search was completed by two authors independently, differences were resolved by discussion.

### Screen studies

The year of publication of studies was restricted from 2000 till present. The inclusion criteria included original clinical studies, articles published in English, TOF patients without endocardial pad defect or other complications, comparison between TOF patients undergoing TAP repair with or without monocusp valve reconstruction, and at least one of the perioperative outcomes stated above was investigated. Studies were excluded for the following criteria: (1) articles published in a language other than English, (2) not an original clinical study, (3) in vitro studies or animal studies, (4) not TOF patients but patients with other diseases undergoing TAP repair, (5) non-comparative study reporting outcomes of patients undergoing TAP repair with or without monocusp valve reconstruction only, (6) conference abstract without a full-text, (7) lack of reported target perioperative outcomes as set out by this study, (8) studies of adult cases (mean age ≥ 18). The sample size was not taken into consideration when screening for eligible studies. This step was completed by all authors except two who were responsible for searching studies. They first decided which literatures needed to screen full-text, then assessed independently. Final inclusion was determined by consensus.

### Data extraction

Data on patient baseline characteristics and perioperative outcomes were extracted from the included studies by two authors independently and repeated twice to ensure the veracity of data. The following data were extracted: study characteristics (including first author, publication year, study design, and sample size), patient baseline characteristics (including gender, age, weight), and all reported target perioperative outcomes stated above. Data was extracted into Excel form by two authors independently, differences would be resolved by consensus, the third author would intervene if necessary.

### Bias analysis and quality assessment

The risk of bias was assessed for all the included studies, in which retrospective cohort studies were assessed using the Cochrane’s Tool to Assess Risk of Bias in Cohort Studies (see methods.cochrane.org), which assesses studies from eight aspects, including subject selection, assessment of exposure, confidence in baseline, confounding variables, prognostic factors, outcome assessment, follow-up, and co-intervention. Randomized controlled trials were assessed using the Cochrane tool for Risk of Bias Assessment (see methods.cochrane.org), which assesses studies based on randomization, assignment, blinding of participants and personnel, blinding of outcome assessment, available outcome data, and selection of reported results [10]. Publication bias was evaluated by funnel plots. The quality of outcomes was assessed using the GRADE, and divided into four levels including high, moderate, low and very low.

### Data analysis

Dichotomous variables were described as frequencies with absolute numbers, and continuous variables were presented as means with standard deviations or medians with ranges. The meta-analysis, forest plots, and funnel plots were drawn using Review Manager 5.3. The Mantel–Haenszel model was used to analyze dichotomous variables, and the inverse variance model was used to analyze continuous variables. Results of dichotomous variables were presented as odds ratios (OR) with 95% confidence intervals (CI), whereas results of continuous variables were presented as the mean difference with 95% CI. *Chi*^2^, degree of freedom, and *I*^2^ were used for heterogeneity test between studies. A random-effects model was firstly used, if *I*^2^ was no bigger than 50% and *p*-value of *Chi*^2^ demonstrated no statistic significance, a fixed-effects model would be used furtherly, while a random-effects model was used if either the *p-*value of *Chi*^2^ was significant or the *I*^2^ > 50%. A *p-*value of ≤ 0.05 was considered statistically significant.

## Results

### Study selection

**Fig. 1:**
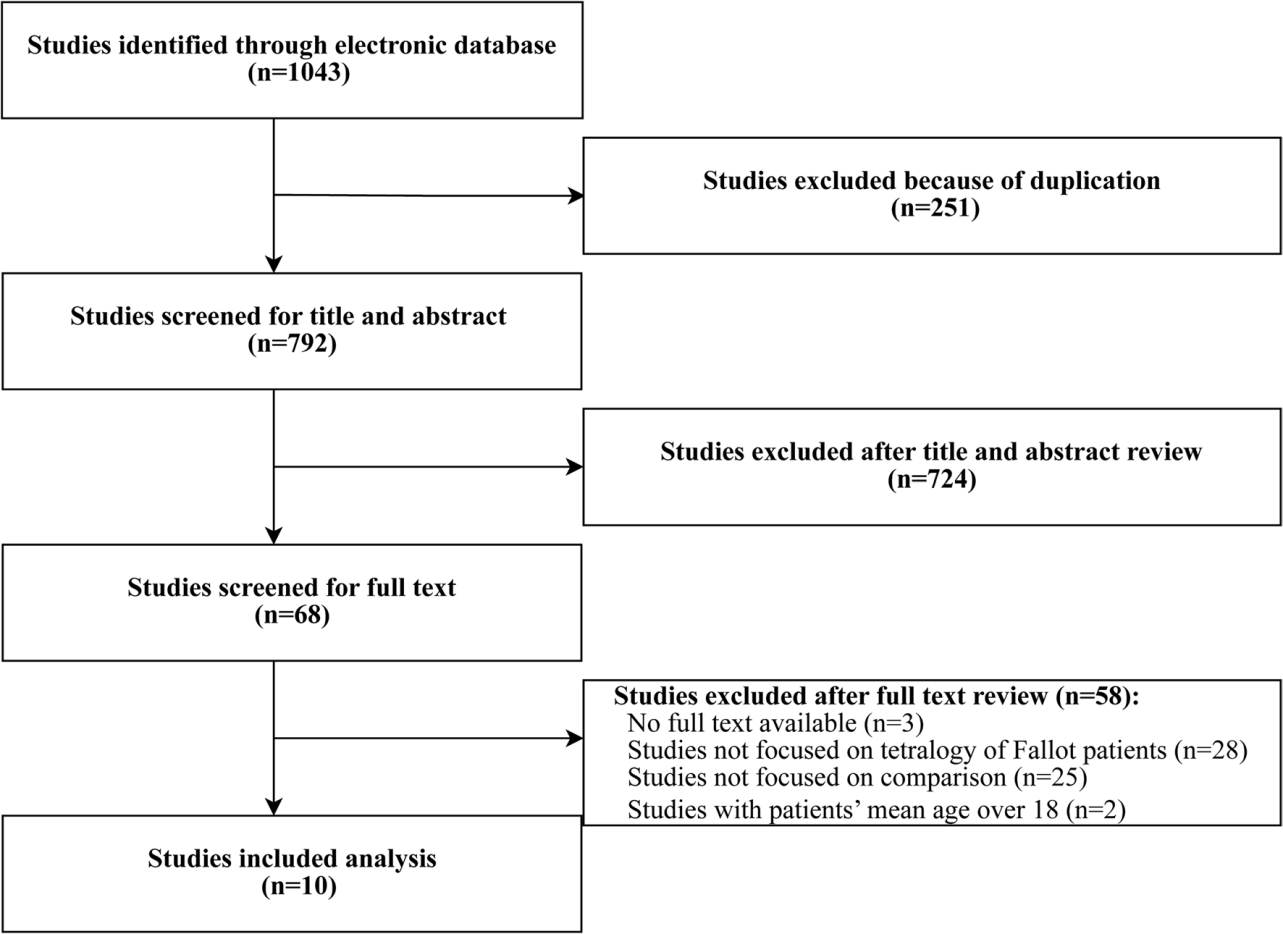
Flow chart of search results and study selection

The initial literature search yielded 792 articles after removing duplication. Upon reviewing titles and abstracts, 68 studies remained and full-text articles were obtained. Of these, 3 studies were excluded as these were conference or meeting summary, whereas 55studies did not fulfill our study criteria, given that studied patients were either not of TOF only, or no comparison was made between the outcomes of patients undergoing TAP repair with or without monocusp valve reconstruction, or patients’ mean age over 18 years old. Finally, 10 articles were included in our analysis [11–20] (Fig. 1), which consisted of 2 randomized controlled trials (RCT) [12, 19] while the others were retrospective cohort studies [11, 13–18, 20].

### Study characteristics

**Fig. 2:**
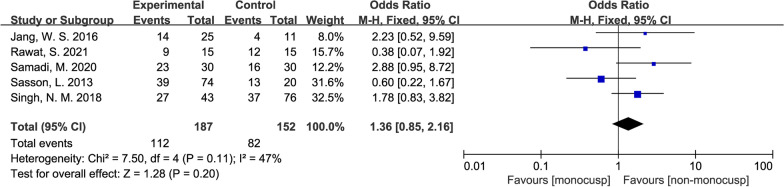
Forest plot demonstrating gender of monocusp group and non-monocusp group

**Fig. 3:**
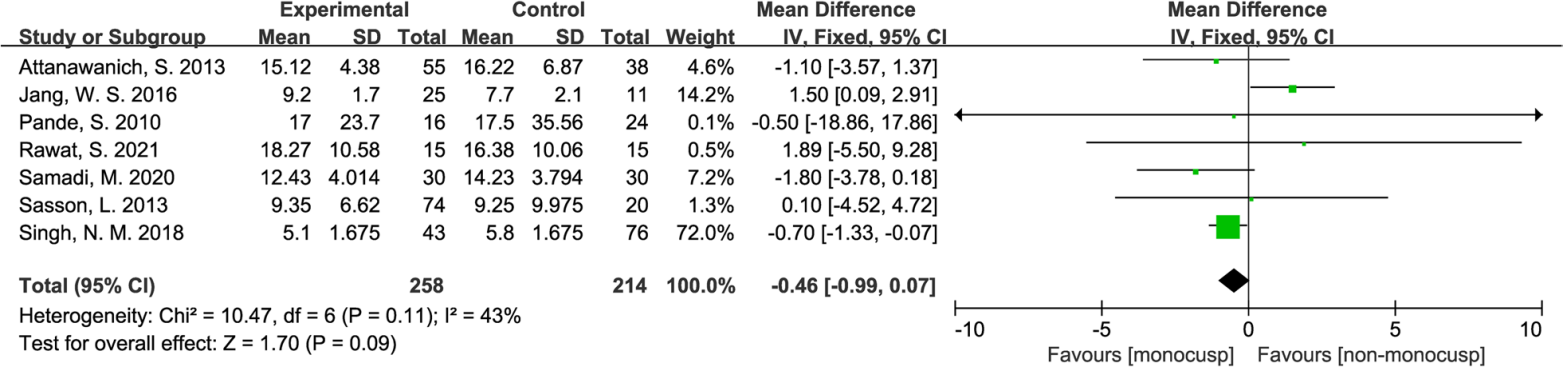
Forest plot demonstrating mean age at operation of monocusp group and non-monocusp group

**Fig. 4:**
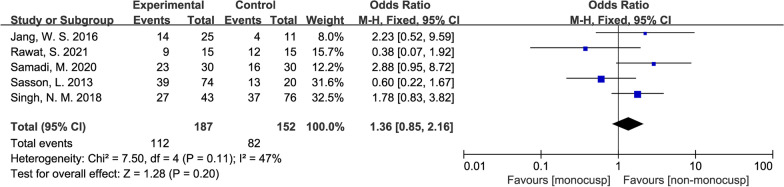
Forest plot demonstrating mean weight at operation of monocusp group and non-monocusp group

Baseline characteristics were variably reported in these 10 studies. Only 5 studies [12, 15, 18–20] reported gender ratio in both the study group and the control group, other studies only reported the number of patients in each group. The mean age at operation was reported in 8 studies [12, 14–20], and the mean body weight was reported in 7 studies [12, 15–20]. The 10 included studies formed a pool of 661 TOF patients in total. Of these, 349 patients underwent TAP repair with monocusp valve reconstruction (monocusp group) and 312 patients underwent TAP repair without monocusp valve reconstruction (non-monocusp group). The gender ratio of male to female was 1.5:1 (112 males, 75 females) in the monocusp group compared to 1:1 (82 males, 70 females) in the non-monocusp group. The specific gender of 332 patients was not reported (Table 1). There was no significant difference between the two groups in patient baseline characteristics according to forest plots (Figs. 2, 3, 4).

**Table 1:** Baseline characteristics of included studies

Study	Study design	Patient number	Sex ratio (male/female)	Age at operation (months)	Weight at operation (kg)
Attanawanich, S. 2013 [17]	Retrospective cohort study				
Monocusp valve		55	–	48.33 ± 24.17	15.12 ± 4.38
Non-monocusp valve		38	–	65.05 ± 30.64	16.22 ± 6.87
Aydın, S. 2018 [13]	Retrospective cohort study				
Monocusp valve		15	–	–	–
Non-monocusp valve		20	–	–	–
Ismail, S. R. 2010 [11]	Retrospective cohort study				–
Monocusp valve		16	–	–	–
Non-monocusp valve		48	–	–	–
Jang, W. S. 2016 [20]	Retrospective cohort study				
Monocusp valve		25	14/11	13.7 ± 6.5	9.2 ± 1.7
Non-monocusp valve		11	4/7	9.7 ± 5.5	7.7 ± 2.1
Pande, S. 2010 [16]	Retrospective cohort study				
Monocusp valve		16	–	90.0 ± 165.33	17.0 ± 23.7
Non-monocusp valve		24	–	132.0 ± 302.22	17.5 ± 35.56
Rawat, S. 2021 [19]	Randomized controlled trial				
Monocusp valve		15	9/6	94.92 ± 78.24	18.27 ± 10.58
Non-monocusp valve		15	12/3	72.72 ± 66.0	16.38 ± 10.06
Samadi, M. 2020 [12]	Randomized controlled trial				
Monocusp valve		30	23/7	36.0 ± 32.88	12.43 ± 4.014
Non-monocusp valve		30	16/14	48.0 ± 27.108	14.23 ± 3.794
Sasson, L. 2013 [18]	Retrospective cohort study				
Monocusp valve		74	39/35	20.5 ± 32.67	9.35 ± 6.62
Non-monocusp valve		20	13/7	27.0 ± 47.0	9.25 ± 9.975
Sayyed, E.H.N. 2016 [14]	Retrospective cohort study				
Monocusp valve		60	–	127.8 ± 90.6	5.1 ± 1.675
Non-monocusp valve		30	–	114.0 ± 67.2	5.8 ± 1.675
Singh, N. M. 2018 [15]	Retrospective cohort study				
Monocusp valve		43	27/16	4.57 ± 12.18	–
Non-monocusp valve		76	37/39	4.78 ± 17.15	–

### Bias analysis and outcomes assessment

The assessment of 8 cohort studies by the Tool to Assess Risk of Bias in Cohort Studies and 2 RCTs by the Cochrane tool for Risk of Bias Assessment are carried out (Additional file 1: Table S1). According to the funnel plots, publication bias of all outcomes was assessed, and significant publication bias has been described in ‘summary of findings’ of GRADE. Furthermore, findings from GRADE showed that the primary outcome (perioperative mortality) had a low quality of evidence; two of the secondary outcomes (ICU stay and moderate or severe pulmonary regurgitation in the perioperative period) had a low quality of evidence; other secondary outcomes were however very low in the quality of evidence (Additional file 2: Table S2). PRISMA checklist could be found in supplementary material (Additional file 3: Table S3).

### Outcomes analysis

#### Perioperative mortality

**Fig. 5:**
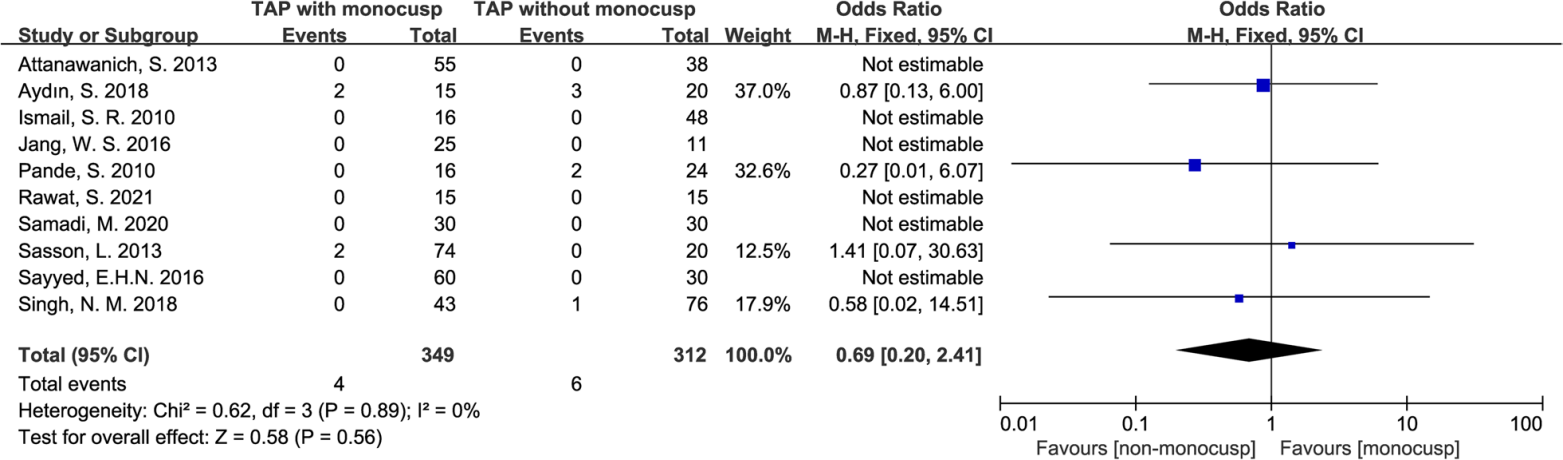
Forest plot demonstrating perioperative mortality between monocusp group and non-monocusp group

There was 1.1% (4/349) perioperative death in the monocusp group compared with 1.9% (46/312) in the non-monocusp group, which was NOT significantly different between the two groups (OR = 0.69, 95% CI 0.20–2.41, *p* = 0.58), though forest plot showed less mortality in monocusp group. Heterogeneity assessment showed an *I*^2^ value of 0%, and *p-*value of *Chi*^2^ was 0.89, indicating no significant heterogeneity in this analysis and thus, a fixed-effects model was used (Fig. 5).

#### Cardiopulmonary bypass (CPB) time

**Fig. 6:**
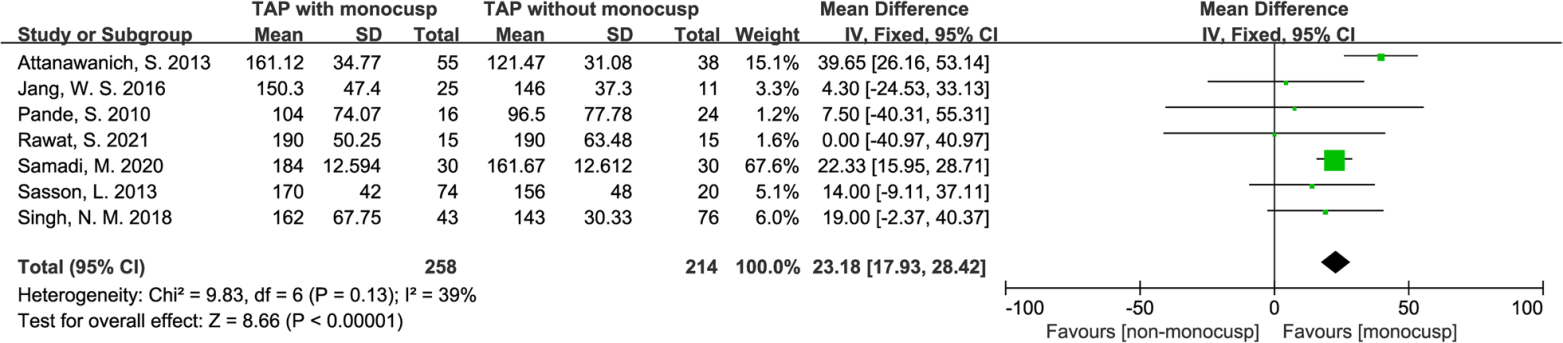
Forest plot demonstrating cardiopulmonary bypass time between monocusp group and non-monocusp group

There was a significant difference in the perioperative cardiopulmonary bypass time between the two groups, with the standard mean difference of 23.18 (minute, 95% CI 17.93–28.42, *p* < 0.00001). A total of 7 studies [12, 15–20] reported this outcome, with 258 patients in the monocusp group and 214 patients in the non-monocusp group for the analysis. Forest plot demonstrated the length of CPB is longer in monocusp group. For this analysis, the *I*^*2*^ value was 39% and *p-*value of *Chi*^2^ was 0.13, indicating no significant heterogeneity in this analysis and thus, a fixed-effects model was used (Fig. 6).

#### Aortic cross-clamp time

**Fig. 7:**
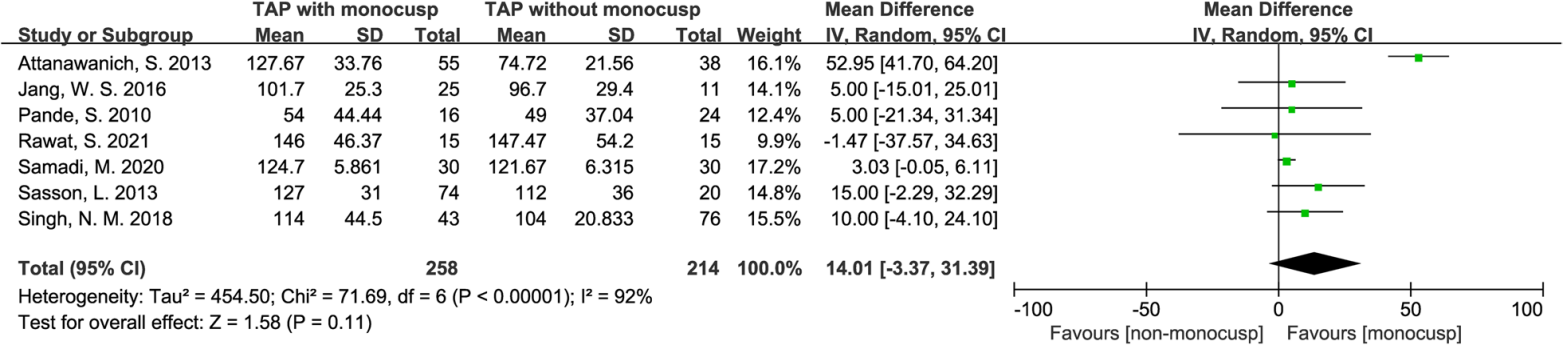
Forest plot demonstrating aortic cross-clamp time between monocusp group and non-monocusp group

A significant difference in the perioperative aortic cross-clamp time between the two groups was NOT demonstrated with the standard mean difference of 14.01 (minute, 95% CI − 3.37–31.39, *p* = 0.11), while forest plot demonstrated the length of cross-clamp time is longer in monocusp group. Data were extracted from 7 studies [12, 15–20], with 258 patients in the monocusp group and 214 in the non-monocusp group for the analysis. *I*^2^ value was 92% and *p-*value of *Chi*^2^ was < 0.00001, which indicated significant heterogeneity in this analysis, thus, a random-effects model was used (Fig. 7).

#### Ventilation duration

**Fig. 8:**
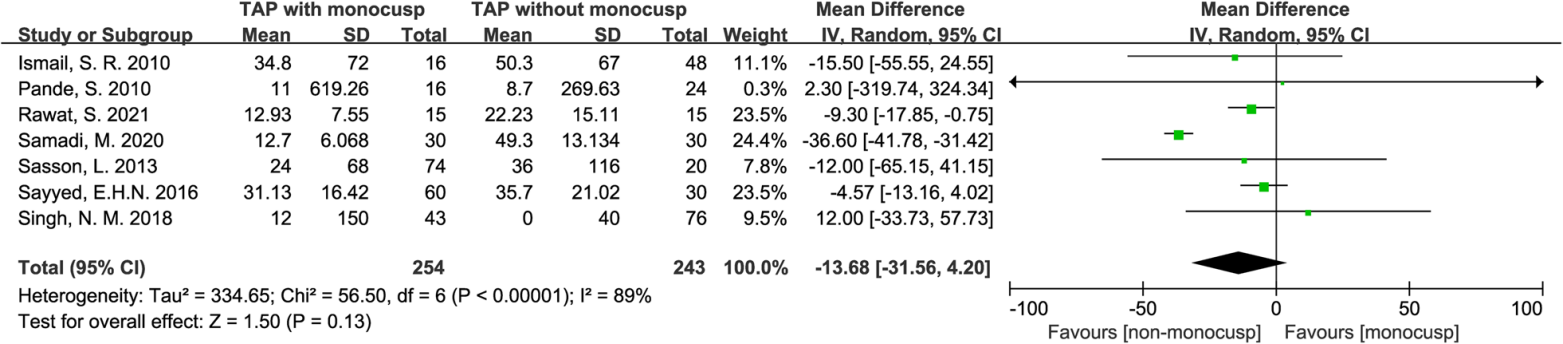
Forest plot demonstrating ventilation duration between monocusp group and non-monocusp group

There was NO significant difference in the perioperative ventilation duration between the two groups with the standard mean difference of − 13.68 (hour, 95% CI − 31.56–4.20, *p* = 0.13), although the forest plot revealed that ventilation duration in monocusp group was shorter. Data were obtained from 7 studies [11, 12, 14–16, 18, 19] and comprised of 254 patients in the monocusp group and 243 in the non-monocusp group for the analysis. An *I*^2^ value of 89% indicated significant heterogeneity in this analysis. Thus, a random-effects model was used (Fig. 8).

#### Length of stay in the intensive care unit

**Fig. 9:**
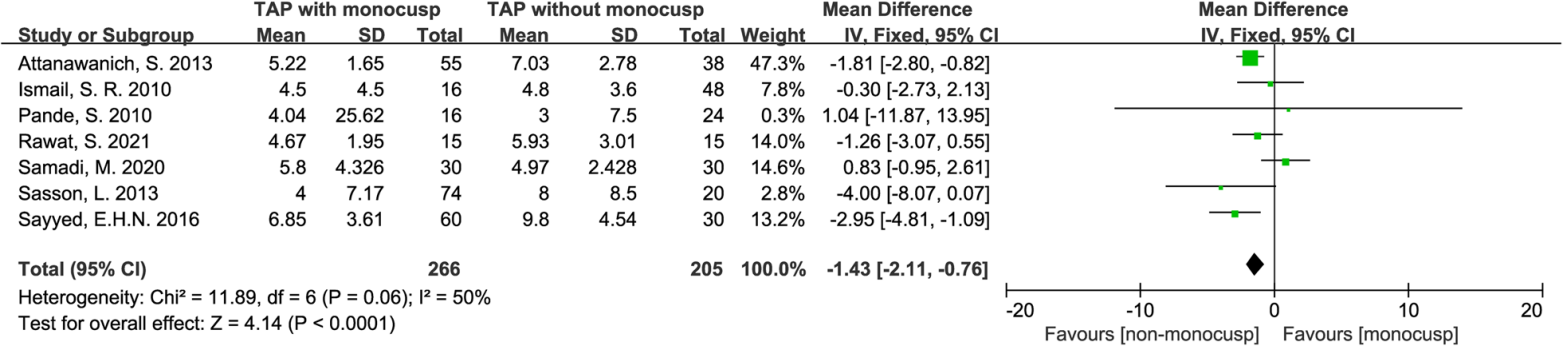
Forest plot demonstrating length of stay in the Intensive Care Unit between monocusp group and non-monocusp group

There was a significant difference in the length of stay in the Intensive Care Unit (ICU) between the two groups with the standard mean difference of − 1.43 (day, 95% CI − 2.11–0.76, *p* < 0.0001). Forest plot demonstrated the length of ICU stay was significantly shorter in monocusp. Data were obtained from 7 studies [11, 12, 14, 16–19] and comprised of 266 patients in the monocusp group and 205 patients in the non-monocusp group for the analysis. *I*^2^ value of 50% and *p-*value of *Chi*^2^ of 0.06 indicated no significant heterogeneity in this analysis, and thus, a fix-effects model was used (Fig. 9).

#### Length of stay in the hospital

**Fig. 10:**
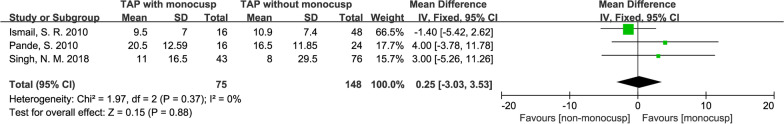
Forest plot demonstrating length of stay in the hospital between monocusp group and non-monocusp group

NO significant difference was demonstrated in the length of stay in the hospital between the two groups with the standard mean difference of 0.25 (day, 95% CI − 3.03–3.53, *p* = 0.88). Forest plot also revealed approximately the same length of hospital stay of two groups. Data were extracted from only 3 studies [11, 15, 16] including 75 patients in the monocusp group and 148 patients in the non-monocusp group for the analysis. An *I*^2^ value of 0% indicated no significant heterogeneity in this analysis, and thus, a fixed-effects model was used (Fig. 10).

#### Perioperative right ventricular outflow tract pressure gradient

**Fig. 11:**
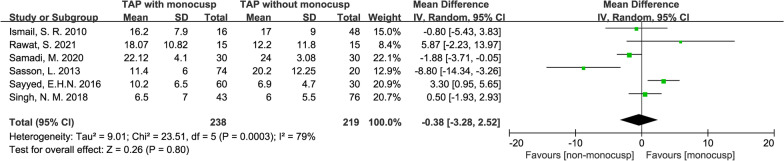
Forest plot demonstrating perioperative right ventricular outflow tract pressure gradient between monocusp group and non-monocusp group

There was NO significant difference in the perioperative ROVT pressure gradient between the two groups with the standard mean difference of − 0.38 (mmHg, 95% CI − 3.28–2.52, *p* = 0.80), and forest plot also demonstrated similar RVOT pressure in these two groups. Data were obtained from 6 studies [11, 12, 14, 15, 18, 19] including 238 patients in the monocusp group and 219 patients in the non-monocusp group for the analysis. An *I*^2^ value of 79% and *p*-value of *Chi*^2^ of 0.0003 indicated significant heterogeneity in this analysis, and thus, a random-effects model was used (Fig. 11).

#### Moderate or severe pulmonary regurgitation in the perioperative period

**Fig. 12:**
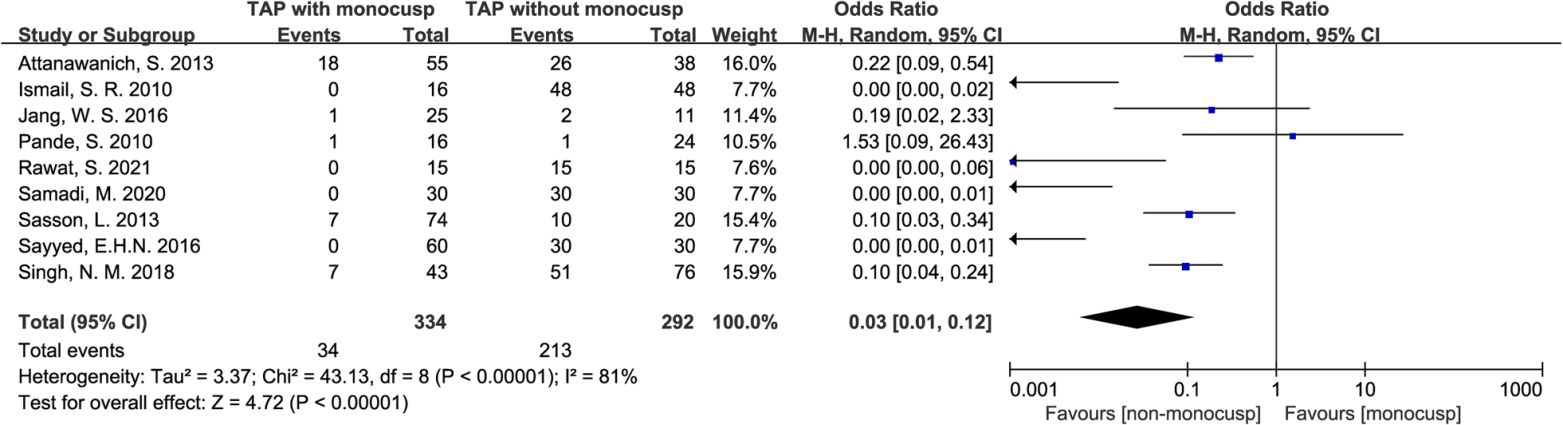
Forest plot demonstrating moderate or severe pulmonary regurgitation in the perioperative period between monocusp group and non-monocusp group

A total of 10% (34/334) of patients in the monocusp group developed moderate or severe perioperative PR compared with 72.9% (213/292) in the non-monocusp group, which was significantly different between the two groups (OR = 0.03, 95% CI 0.01–0.12, *p* < 0.00001). Forest plot also demonstrated during perioperative period, there were less patients with moderate or severe PR in monocusp group. Data were obtained from 9 studies [11, 12, 14–20] comprised of 334 patients in the monocusp group and 292 patients in the non-monocusp for the analysis. The *p*-value of *Chi*^2^ was < 0.00001 and *I*^2^ value was 81%, which indicated significant heterogeneity in this analysis, a random-effects model was used (Fig. 12).

## Discussion

### Discussion of current results and advantages

Our analyses have demonstrated several differences in the perioperative outcomes comparing TOF patients undergoing TAP repair with and without monocusp valve reconstruction. TAP repair with monocusp valve reconstruction was associated with significant benefits to the mean length of ICU stay and the degree of perioperative PR, although inserting a monocusp means more complicated procedure and longer length of CPB time and aortic cross-clamp time. In particular, several studies [4, 9, 12, 14, 15, 18] have reported a prominent effect of monocusp valve reconstruction in reducing the degree of perioperative PR, which may then confer to improvements of respiratory symptoms and quality of life. Although the cost of hospitalization has not been investigated by any studies, the evidence of the shorter length of stay in the ICU may potentially reduce the healthcare cost in patients undergoing TAP repair with monocusp valve reconstruction. Analysis of other clinical outcomes including perioperative mortality, ventilation duration, hospital stay, and perioperative right ventricular outflow tract pressure gradient demonstrated some minor superiority in the monocusp group when compared with those without monocusp valve reconstruction, the differences however was not statistically significant. In general, though early mortality and most secondary outcomed revealed no significant difference through statistical analysis, the monocusp seems to have a potential effect to benefit TOF patients with severe RVOT stenosis.To reduce the risk of perioperative PR, TAP repair should be avoided to preserve the pulmonary valve [9]. This may be feasible in the repair of early TOF [21]. However, TOF patients with a severe deformity of pulmonary artery annulus leading to severe RVOT obstruction would necessitate a TAP repair. Our analyses have shown the advantages of TAP repair with monocusp valve reconstruction in the perioperative in-hospital period. We have excluded investigating long-term postoperative outcomes, given that very few studies to date have compared the long-term outcomes of TAP repair with or without monocusp valve reconstruction in patients with TOF, and the limited data available would not be sufficient for a meta-analysis. Besides, some studies have shown a low mortality rate at two or three years after surgery for TOF [4, 22, 23] which may result in a higher rate of loss to follow-up, leading to lower quality of long-term outcome data.

### Limitation

One noteworthy limitation in our meta-analysis was that materials of the monocusp were not brought into study. For all included studies, 3 declared that they used autologous pericardium to form monocusp, [13, 16, 17] another 3 used monocusp of polytetrafluoroethylene (PTFE) [18–20], other studies used more than one type of material or did not explain specific material of monocusp. Besides, we have noticed that 1 of the included studies has compared outcomes between TAP with autologous pericardium monocusp and PTFE monocusp, which showed no significant difference in outcomes at 3-year follow-up [14]. A few studies used a new technique named “Right atrial appendage (RAA) valve” to prevent postoperative PR, which showed significant improvement compared to conventional TAP repair [24, 25]. More studies about RAA valve are needed to evaluate whether it is possible to replace previous techniques. Limited by small quantity of included studies and patients, a subgroup-analysis of monocusp materials is difficult to carry out. Another limitation was that TOF patients usually operated in 6 to 24 months, however, to ensure sufficient sample size, some studies with mean operation age nearly ten were included in this meta-analysis [14, 16, 19]. Though analysis of baseline demonstrated no significant difference, operation age might still influence the veracity of the study. The last limitation we have found was that not all studies reported specific baseline characteristics, which may affect accuracy of this meta-analysis. Because of most of our included studies were cohort studies, the quality of outcomes was not high enough, which may also influence analysis results as a possible factor.

Our meta-analysis represents the first to combine clinical studies to address a currently controversial practice concerning monocusp valve reconstruction during TAP repair in patients with TOF. However, our study was limited by the lack of data and large-scale high-quality studies, which therefore, warrants a large, multicenter, randomized, prospective study to validate our findings.

## Conclusion

Transannular patch repair with monocusp valve reconstruction has significant advantages on decreasing the length of ICU stay and reducing degree of perioperative PR of TOF patients compared to TAP without monocusp, but the study demonstrated no significant benefit on perioperative mortality. However, TAP with monocusp seems to have a potential effect to benefit TOF patients with severe RVOT stenosis. Further well-designed, multicenter, randomized, prospective studies focusing on perioperative outcomes and postoperative differences based on long-term follow-up between TAP repair with and without monocusp valve reconstruction are needed.

## Supplementary Information

**Additional file 1: Table S1.** Bias assessment of included studies

**Additional file 2: Table S2.** Quality of results assessed by GRADE

**Additional file 3: Table S3. **PRISMA checklist.

## Data Availability

All relevant data has been provided in figures, tables and text.

## Abbreviations

CI: Confidence interval
CPB: Cardiopulmonary bypass
GRADE: Grading of recommendations assessment, development and evaluation
ICU: Intensive care unit
OR: Odds ratio
PRISMA: Preferred reporting items in systematic reviews and meta-analysis
RAA: Right atrial appendage
RCT: Randomized controlled trial
RVH: Right ventricular hypertrophy
RVOT: Right ventricular outflow tract
TAP: Transannular patch
TOF: Tetralogy of Fallot
VSD: Ventricular septal defect
