# Supplementary material for: Transannular patch repair of tetralogy of Fallot with or without monocusp valve reconstruction: a meta-analysis
Source: BMC Surg. 2022 Jan 16;22:18. doi: 10.1186/s12893-022-01474-6 (PMC8762931; doi:10.1186/s12893-022-01474-6)
Supplement: Supplementary file 1 — Additional file 1: Table S1. Bias assessment of included studies [file 12893_2022_1474_MOESM1_ESM.pdf]

Risk of bias summary: assessment of retrospective cohort studies

	Retrospective cohort studies							
	Attanawanich, S. 2013[17]	Aydın, S. 2018[13]	Ismail, S. R. 2010[11]	Jang, W. S. 2016[20]	Pande, S. 2010[16]	Sasson, L. 2013[18]	Sayyed, E.H.N. 2016[14]	Singh, N. M. 2018[15]
Was selection of exposed and nonexposed cohorts drawn from the same population?	Definitely yes	Definitely yes	Definitely no	Definitely yes	Definitely yes	Definitely yes	Definitely yes	Probably yes
Can we be confident in the assessment of exposure?	Definitely yes	Probably yes	Probably yes	Definitely yes	Definitely yes	Definitely yes	Definitely yes	Definitely yes
Can we be confident that the outcome of interest was not present at start of study?	Definitely yes	Probably yes	Definitely yes	Definitely yes	Definitely yes	Definitely yes	Definitely yes	Definitely yes
Did the study match exposed and unexposed for all variables that are associated with the outcome of interest or did the statistical analysis adjust for these prognostic variables?	Definitely yes	Probably yes	Probably yes	Definitely yes	Probably yes	Probably yes	Probably yes	Probably yes
Can we be confident in the assessment of the presence of prognostic factors?	Definitely yes	Probably yes	Probably yes	Definitely yes	Definitely yes	Probably yes	Definitely yes	Probably yes
Can we be confident in the assessment of outcome?	Definitely yes	Probably yes	Probably yes	Definitely yes	Definitely yes	Definitely yes	Definitely yes	Probably yes
Was the followup of cohorts adequate?	Definitely yes	Probably yes	Definitely yes	Definitely yes	Definitely yes	Probably yes	Probably yes	Definitely yes
Were co-interventions similar between groups?	Definitely yes	Definitely yes	Definitely yes	Definitely yes	Definitely yes	Definitely yes	Definitely yes	Definitely yes

Risk of bias summary: assessment of RCTs

	RCTs	
	Rawat, S. 2021	Samadi, M. 2020
Random sequence generation<br>(selection bias)	Low risk	Low risk
Allocation concealment (selection bias)	Low risk	Low risk
Blinding of participants and personnel (performance bias)	Unclear risk	Unclear risk
Blinding of outcome assessment<br>(detection bias)	Unclear risk	Unclear risk
Incomplete outcome data<br>(attrition bias)	Low risk	Low risk
Selective reporting (reporting bias)	Low risk	Low risk
Other bias	Low risk	Low risk
