# Supplementary material for: Transannular patch repair of tetralogy of Fallot with or without monocusp valve reconstruction: a meta-analysis
Source: BMC Surg. 2022 Jan 16;22:18. doi: 10.1186/s12893-022-01474-6 (PMC8762931; doi:10.1186/s12893-022-01474-6)
Supplement: Supplementary file 2 — Additional file 2: Table S2. Quality of results assessed by GRADE [file 12893_2022_1474_MOESM2_ESM.pdf]

TAP with monocusp compared to TAP without monocusp for TOF patients with severe stenosis of RVOT

Patient or population: TOF patients with severe stenosis of RVOT
Settings:
Intervention: TAP with monocusp
Comparison: TAP without monocusp

Outcomes	Illustrative comparative risks* (95% CI)		Relative effect (95% CI)	No of Participants (studies)	Quality of the evidence (GRADE)	Comments
	Assumed risk	Corresponding risk				
	**TAP without monocusp**	**TAP with monocusp**				
**Early death/ Inpatient mortality**	**Study population**		**OR 0.69** (0.2 to 2.41)	661 (10 studies)	⊕⊕⊝⊝ **low**	
	**19 per 1000**	**13 per 1000** (4 to 45)				
	**Moderate**					
	**0 per 1000**	**0 per 1000** (0 to 0)				

*The basis for the **assumed risk** (e.g. the median control group risk across studies) is provided in footnotes. The **corresponding risk** (and its 95% confidence interval) is based on the assumed risk in the comparison group and the **relative effect** of the intervention (and its 95% CI).

CI: Confidence interval; **OR:** Odds ratio;

GRADE Working Group grades of evidence
High quality: Further research is very unlikely to change our confidence in the estimate of effect.
Moderate quality: Further research is likely to have an important impact on our confidence in the estimate of effect and may change the estimate.
Low quality: Further research is very likely to have an important impact on our confidence in the estimate of effect and is likely to change the estimate.
Very low quality: We are very uncertain about the estimate.

TAP with monocusp compared to TAP without monocusp for TOF patients with severe stenosis of RVOT

Patient or population: TOF patients with severe stenosis of RVOT
Settings:
Intervention: TAP with monocusp
Comparison: TAP without monocusp

Outcomes	Illustrative comparative risks* (95% CI)		Relative effect (95% CI)	No of Participants (studies)	Quality of the evidence (GRADE)	Comments
	Assumed risk	Corresponding risk				
	**TAP without monocusp**	**TAP with monocusp**				
**Cardiopulmonary bypass time (min)**		The mean cardiopulmonary bypass time (min) in the intervention groups was **23.18 higher** (17.93 to 28.42 higher)		472 (7 studies)	⊕⊝⊝⊝ **very low**[1,2]	
**Aortic cross-clamp time (min)**		The mean aortic cross-clamp time (min) in the intervention groups was **14.01 higher** (3.37 lower to 31.39 higher)		472 (7 studies)	⊕⊝⊝⊝ **very low**[1,3]	
**Ventilation duration (h)**		The mean ventilation duration (h) in the intervention groups was **13.68 lower** (31.56 lower to 4.2 higher)		497 (7 studies)	⊕⊝⊝⊝ **very low**[1,3]	
**ICU stay (d)**		The mean icu stay (d) in the intervention groups was **1.43 lower** (2.11 to 0.76 lower)		471 (7 studies)	⊕⊕⊝⊝ **low**	
**Hospital stay (d)**		The mean hospital stay (d) in the intervention groups was **0.25 higher** (3.03 lower to 3.53 higher)		223 (3 studies)	⊕⊝⊝⊝ **very low**[4]	
**Perioperative RVOT pressure gradient (mmHg)**		The mean perioperative rvot pressure gradient (mmhg) in the intervention groups was **0.38 lower** (3.28 lower to 2.52 higher)		457 (6 studies)	⊕⊝⊝⊝ **very low**[3]	
**Moderate or severe pulmonary regurgitation in perioperative period**	**Study population**		**OR 0.03** (0.01 to 0.12)	626 (9 studies)	⊕⊕⊝⊝ **low**[3,5]	
	**729 per 1000**	**75 per 1000** (26 to 244)				
	**Moderate**					
	**684 per 1000**	**61 per 1000** (21 to 206)				

*The basis for the **assumed risk** (e.g. the median control group risk across studies) is provided in footnotes. The **corresponding risk** (and its 95% confidence interval) is based on the assumed risk in the comparison group and the **relative effect** of the intervention (and its 95% CI).

CI: Confidence interval; **OR:** Odds ratio;

GRADE Working Group grades of evidence
High quality: Further research is very unlikely to change our confidence in the estimate of effect.
Moderate quality: Further research is likely to have an important impact on our confidence in the estimate of effect and may change the estimate.
Low quality: Further research is very likely to have an important impact on our confidence in the estimate of effect and is likely to change the estimate.
Very low quality: We are very uncertain about the estimate.

[1] The width of confidence interval is wide
[2] funnel demonstrated significant publication bias
[3] I2 over 75%, which suggested significant heterogeneity
[4] No explanation was provided
[5] RR=0.14
